# Has carbohydrate-restriction been forgotten as a treatment for diabetes mellitus? A perspective on the ACCORD study design

**DOI:** 10.1186/1743-7075-5-10

**Published:** 2008-04-09

**Authors:** Eric C Westman, Mary C Vernon

**Affiliations:** 1 Department of Medicine, Duke University Medical Center, Durham, NC, USA; 2 Private Practice, Lawrence and Lenexa, KS, USA

## Abstract

Prior to the discovery of medical treatment for diabetes, carbohydrate-restriction was the predominant treatment recommendation to treat diabetes mellitus. In this commentary we argue that carbohydrate-restriction should be reincorporated into contemporary treatment studies for diabetes mellitus.

## Introduction

In the early 20^th ^century, before any medications were available for the treatment of diabetes mellitus, experts recommended dietary carbohydrate-restriction [1,2]. The dietary recommendation for diabetes in a prominent internal medicine textbook from 1923 was 75% fat, 17% protein, 6% alcohol and only 2% carbohydrate [3]. The recommended total daily energy intake was 1,795 Calories per day. After the discovery of insulin and oral hypoglycemic medications, experts gradually changed the dietary recommendations to include more carbohydrate intake because most experts reasoned that the medications could be used to keep the glucose in control.

The NIH NHLBI Action to Control Cardiovascular Risk in Diabetes (ACCORD) group recently announced termination of the intensive insulin therapy arm of their study after an interim analysis showed that mortality was significantly higher in this group than in the other two less intensive glucose control groups [4,5]. Because lead investigators from the ACCORD trial and other experts have stated how unexpected this finding was, and have suggested that the concept of normal glucose control among patients with type 2 diabetes may not be desirable, we feel compelled to provide an alternative view.

## Discussion

From our perspective of familiarity with dietary carbohydrate-restriction and diabetes, these results are not surprising–in fact, they are predicted. We believe that it is unlikely that the increased mortality was due to the tight glucose control but rather due to the particular method for trying to achieve it. When *high carbohydrate diets *are consumed and intensive medication therapy is used to "cover the carbohydrate," it is very difficult to achieve normal glycemic control *without hypoglycemic reactions*. In our clinical practices, we frequently see individuals who are instructed to eat high carbohydrate diets and use intensive injectable hypoglycemic therapy, and they are susceptible to hypoglycemic reactions. Severe hypoglycemic reactions are associated with an increased morbidity and mortality [6].

There are other ways to improve glycemic control without the risk of hypoglycemic reactions; one of these is carbohydrate-restriction. Carbohydrate-restriction makes pathophysiological sense because type 2 diabetes is, in essence, a case of *carbohydrate intolerance*. We have observed that the same patients who have hypoglycemic reactions with high carbohydrate diets and aggressive medication therapy no longer have hypoglycemic reactions with carbohydrate-restriction. Moreover, the continued concerns about carbohydrate-restricted diets have never materialized and recent scientific studies show general health benefits including reduced cardiometabolic risk factors [7-10].

Based on the clinical experience of others, and published clinical trials, we use carbohydrate-restriction in clinical practice for the treatment of diabetes mellitus [11-15]. At the end of our clinic day, we go home thinking, "The clinical improvements are so large and obvious, why don't other doctors understand?" Carbohydrate-restriction is easily grasped by patients: because carbohydrates in the diet raise the blood glucose, and as diabetes is defined by high blood glucose, it makes sense to lower the carbohydrate in the diet. By reducing the carbohydrate in the diet, we have been able to taper patients off as much as 150 units of insulin per day in 8 days, with marked improvement in glycemic control-even normalization of glycemic parameters. Due to the potent effect of carbohydrate restriction in decreasing blood glucose levels, we must reduce the insulin by 50% on the first day of dietary carbohydrate-restriction to avoid hypoglycemia. As the weeks pass, most patients achieve normoglycemia without medication, obese patients lose weight, and patients save money because they are not paying for medications. It is not so far-fetched to predict that these savings will also be passed along to the health care system and self-insured companies because there will be less expenditure on medications and the long-term diabetic complications.

## Conclusion

The inattention to *potent *dietary therapy in all recent major diabetes studies, including the recent ACCORD trial, should not lead us to forget about carbohydrate-restriction as a means to achieve weight loss and glycemic control without hypoglycemia. We urgently need controlled studies comparing the newer "higher-carbohydrate diet with or without medication" approach to the earlier "carbohydrate-restricted diet without medication" approach for type 2 diabetes mellitus. One of the important advantages of carbohydrate-restriction is that there is *no risk *of hypoglycemia if medications are not used. We believe that carbohydrate-restriction has come of age for the treatment of obesity and diabetes mellitus and should be urgently translated from clinical practice to intensive testing in studies relating to mechanism, health services research, and public health.

## Competing interests

ECW has received unrestricted research grant funding from the Robert C. Atkins Foundation. MCV has written a book about the treatment of diabetes with carbohydrate-restriction.
